# Study partner‐reported decline as a predictor of diagnostic progression in underrepresented populations in the Alzheimer’s Disease Neuroimaging Initiative cohort

**DOI:** 10.1002/alz.095111

**Published:** 2025-01-09

**Authors:** Amanda T. Calcetas, Alyssa Arentoft, Hyunjung Lee, Heining Cham, Miriam T. Ashford, Adam Diaz, Adeyinka Ajayi, Omobolanle Ayo, Alicia M. Camuy, Julia E. Culhane, Kaori Kubo Germano, Vanessa A. Guzman, Shaniya Parkins, Sandra Talavera, Monica Rivera Mindt

**Affiliations:** ^1^ Fordham University, New York, NY USA; ^2^ California State University Northridge, Northridge, CA USA; ^3^ Northern California Institute for Research and Education (NCIRE), San Francisco, CA USA; ^4^ Veterans Affairs Medical Center, San Francisco, CA USA; ^5^ Icahn School of Medicine at Mount Sinai, New York, NY USA

## Abstract

**Background:**

It is imperative to identify underrepresented populations (URPs) at risk for progression to Mild Cognitive Impairment (MCI) and dementia due to Alzheimer’s disease (AD), yet substantial heterogeneity exists in the presentation and risk of AD among URPs. Previous research with predominantly non‐Latinx White participants indicates early functional decline is associated with increased risk and can be effectively evaluated by participants and study partners (SPs). This study aims to understand the association between subjective functional/cognitive decline and objectively‐measured cognitive decline in URPs. Specifically, we hypothesize that SP‐reported decline predicts diagnostic progression over time better than participant‐reported decline in URPs.

**Method:**

A sample of 283 Alzheimer’s Disease Neuroimaging Initiative (ADNI) participant‐study partner dyads (M_age_69.6 at baseline (±8.1), 62.1% female, 48.4% Black/African American, 29.0% Latinx, 15.5% Asian American, 0.4% Native Hawaiian/Pacific Islander, 1.4% American Indian/Alaska Native, and 8.8% of more than one race), completed the Everyday Cognition Questionnaire (ECog), which assesses real‐world functioning related to specific neuropsychological domains, and received consensus neurocognitive diagnoses. An autoregressive, cross‐lagged panel analysis examined whether ECog and diagnostic conversion from cognitively normal to MCI or MCI to AD were concurrently associated, whether participant‐reported ECog predicted future diagnostic conversion, and whether SP‐reported ECog predicted future diagnostic conversion across 3 time points (baseline, 12‐months, and 24‐months).

**Result:**

All autoregressive paths of ECog and diagnostic progression were positive and significant (*p*s < .05). Cross‐lagged paths of SP‐reported ECog more strongly predicted diagnostic progression than participant‐reported ECog, and significantly predicted diagnostic progression from Time 2 (*n* = 130) to Time 3 (*n* = 92) (*b* = 2.03, SE = 0.64, *p* = .002). Cross‐lagged paths of participant‐reported ECog were not a significant predictor of diagnostic progression across three time points (*p*s > .05). Both models including SP‐reported ECog were significantly stronger at predicting diagnostic progression.

**Conclusion:**

Early functional decline reported by SPs may be an independent predictor for cognitive decline in URPs. SPs also more accurately predicted cognitive decline cross‐sectionally and longitudinally than their participant counterparts. Further characterization of the relationships between URP participants and their SPs and their involvement in AD research should be prioritized.

